# DHT causes liver steatosis via transcriptional regulation of SCAP in normal weight female mice

**DOI:** 10.1530/JOE-21-0040

**Published:** 2021-06-01

**Authors:** Tina Seidu, Patrick McWhorter, Jessie Myer, Rabita Alamgir, Nicole Eregha, Dilip Bogle, Taylor Lofton, Carolyn Ecelbarger, Stanley Andrisse

**Affiliations:** 1 Department of Physiology and Biophysics, Howard University College of Medicine, Washington, DC, USA; 2 Department of Chemistry, Youngstown State University, Youngstown, Ohio, USA; 3 Department of Biology, University of Missouri, Columbia, Missouri, USA; 4 Department of Medicine, Georgetown University Medical Center, Washington, DC, USA; 5 Department of Pediatrics, School of Medicine, Johns Hopkins University, Baltimore, Maryland, USA

**Keywords:** hyperandrogenemia or androgen excess, lipogenesis, SREBP, androgen signaling

## Abstract

Hyperandrogenemia (HA) is a hallmark of polycystic ovary syndrome (PCOS) and is an integral element of non-alcoholic fatty liver disease (NALFD) in females. Administering low-dose dihydrotestosterone (DHT) induced a normal weight PCOS-like female mouse model displaying NAFLD. The molecular mechanism of HA-induced NAFLD has not been fully determined. We hypothesized that DHT would regulate hepatic lipid metabolism via increased SREBP1 expression leading to NAFLD. We extracted liver from control and low-dose DHT female mice; and performed histological and biochemical lipid profiles, Western blot, immunoprecipitation, chromatin immunoprecipitation, and real-time quantitative PCR analyses. DHT lowered the 65 kD form of cytosolic SREBP1 in the liver compared to controls. However, DHT did not alter the levels of SREBP2 in the liver. DHT mice displayed increased SCAP protein expression and SCAP-SREBP1 binding compared to controls. DHT mice exhibited increased AR binding to intron-8 of SCAP leading to increased SCAP mRNA compared to controls. FAS mRNA and protein expression was increased in the liver of DHT mice compared to controls. p-ACC levels were unaltered in the liver. Other lipid metabolism pathways were examined in the liver, but no changes were observed. Our findings support evidence that DHT increased *de novo* lipogenic proteins resulting in increased hepatic lipid content via regulation of SREBP1 in the liver. We show that in the presence of DHT, the SCAP-SREBP1 interaction was elevated leading to increased nuclear SREBP1 resulting in increased *de novo* lipogenesis. We propose that the mechanism of action may be increased AR binding to an ARE in SCAP intron-8.

## Introduction

Polycystic ovary syndrome (PCOS) affects 10–20% of reproductive-aged women. Hyperandrogenemia (HA) is a hallmark of PCOS. Impaired insulin signaling in white adipose tissue (WAT) promotes lipolysis leading to increased serum triglycerides and free fatty acids (FFA), thus driving lipid accumulation mainly in the liver (Samuel & Shulman 2016). Previous work has shown that low-dose DHT female mice displayed obesity-independent impaired glucose tolerance, insulin resistance, and increased hepatic gluconeogenesis without increased serum triglycerides or FFAs (Andrisse *et al.* 2017). Interestingly, despite not getting fat, here we show that these low-dose DHT female mice displayed obesity-independent increased hepatic lipid content.

HA is a common endocrinopathy and contributes significantly to increased rates of metabolic syndrome (MetS), including nonalcoholic fatty liver disease (NAFLD) and type 2 diabetes (T2D). NAFLD is characterized by lipid accumulation due to increased FFA uptake, *de novo* lipogenesis (DNL), and/or reduced lipid removal. (Dowman *et al.* 2010, Pettinelli *et al.* 2011, Berlanga *et al.* 2014). The development of hepatic steatosis in PCOS is unique due to the influence of HA and is multi-factorial and complex. Accumulation of serum-derived FFAs and intrahepatic FA manipulate hepatic lipid metabolism, primarily altering lipogenic gene transcription (Berlanga *et al.* 2014).

The method wherein the liver produces endogenous FAs is called DNL. This comprises *de novo* synthesis of FAs via an intricate cytosolic process in which glucose is transformed to acetyl-CoA by glycolysis and the oxidation of pyruvate. Acetyl-CoA carboxylase (ACC1) then alters acetyl-CoA into malonyl-CoA. Lastly, fatty acid synthase (FAS) makes palmitic acid from malonyl-CoA and acetyl-CoA (Fabbrini *et al.* 2010). Depending on the metabolic state, FAs are then processed to TGs and stored or rapidly metabolized.

DNL is controlled mostly at the transcriptional level (Berlanga *et al.* 2014). Nuclear transcription factors (Liver X receptor (LxRα), sterol regulatory element-binding protein 1c (SREBP1c), carbohydrate-responsive element-binding protein (ChREBP), and farnesoid X receptor (FxR) and enzymes ACC1, FAS, and stearoyl CoA desaturase 1 (SCD1)) are all involved. After a meal, plasma glucose and insulin levels are increased, encouraging the stimulation of lipogenesis via the activation of ChREBP and SREBP1c, respectively (Musso *et al.* 2009). Clinically, NAFLD has been linked with the augmented hepatic appearance of several genes involved in DNL (Mitsuyoshi *et al.* 2009). The effect of low-dose DHT on all these genes was examined here with a focus on the effect of DHT on SREBP1 and DNL.

In this project, we utilize a low-dose DHT model that mimics normal-weight PCOS (Andrisse *et al.* 2017, 2018). In this normal weight PCOS model, diet/food intake is not altered thus the increased hepatic lipid accumulation must be either increased by uptake of FAs or increased by endogenous synthesis. Here, we examined the impact of DHT on DNL and other lipid metabolic pathways.

SREBPs traverse the ER membrane, and in reaction to low levels of sterols, the N-terminal domain of SREBPs are proteolytically stimulated, distributed from the membrane, and then translocated to the nucleus whereby stimulating the appearance of genes-controlling lipid metabolism (Hua* et al.* 1996*a*,*b*. This proteolytic initiation involves the serial cleavage of SREBPs at site-1, in the ER lumen, and subsequently, cleavage at site-2, in the ER transmembrane (Hua* et al.* 1996*b*). Serine protease, site-1 protease (S1P), divides the N-terminal and C-terminal areas of SREBPs (Rawson *et al.* 1997). Site-2 protease (S2P) subsequently cleaves and releases the activated N-terminal domain for nuclear translocation (Rawson *et al.* 1997). This proteolytic process is controlled by sterol levels and SREBP cleavage-activating protein (SCAP) (Cheng *et al.* 2015). SCAP is a sterol sensor that is bound to the C-terminal regulatory domains of SREBPs. SCAP synchronizes the cleavage of SREBPs at site-1. SCAP is triggered by low sterol levels and is inhibited by high sterol levels.

Sterol regulatory element-binding protein-1 (SREBP1) is the master regulator of lipogenesis. The active form of SREBP1 translocates to the nucleus, where it regulates the activity of key lipogenic enzymes – fatty acid synthase and acetyl-CoA carboxylase (Moreau *et al.* 2009). Acetyl-CoA carboxylase (ACC) is a complex multifunctional enzyme system which catalyzes the carboxylation of acetyl-CoA to malonyl-CoA, the rate-limiting step in the fatty acid synthesis. The catalytic function of ACCα (ACC1) is regulated by phosphorylation (inactive) and dephosphorylation (active) of targeted serine residues (Sakr *et al.* 2018). FAS is the anabolic enzyme that contains seven unique catalytic sites and mediates the conversion of acetyl-CoA and malonyl-CoA, in the presence of the cofactor NADPH, into long-chain saturated fatty acids, such as palmitate (Sakr *et al.* 2018).

ChREBP is a transcription factor that binds to the carbohydrate-responsive element (Ishii *et al.* 2004). ChREBP is expressed in the liver and is activated by high glucose. ChREBP is critical for the optimal long-term storage of excess carbohydrates as fats. ChREBP acts together with SREBP1c to stimulate lipogenic genes (Dentin *et al.* 2004). Six fatty acid transporter protein (FATP) isoforms have been identified in mammalian cells, which contain a common motif for FA uptake and fatty acyl-CoA synthetase (ACS) function (Doege & Stahl 2006). FATP2 (ASCVL1) and FATP5 (ASCVL6) are highly expressed in the liver. Peroxisome proliferator-activated receptor gamma (PPARγ) is a master transcriptional regulator of adipogenesis and plays an important role in the process of lipid storage (Okamura *et al.* 2010). PPAR alpha (PPARα) and PPARγ have opposing functions in the regulation of fat metabolism; PPARα promotes utilization (beta-oxidation, colloquially referred to as burning fat), while activation of PPARγ promotes storage (fatty acid synthesis). FxR activation reduced the expression of SREBP1c and activated PPARα (Watanabe *et al.* 2004).

AR has traditionally been thought of as only having a nuclear function as a transcription factor. However, several recent studies have shown that AR directly interacts with cytosolic signaling pathways (Baron *et al.* 2004, Andrisse *et al.* 2017). Androgens promoted DNL and lipid uptake via indirect stimulation of a family of transcription factors called sterol regulatory element-binding proteins (SREBPs) (Swinnen *et al.* 1997, Heemers *et al.* 2006). These interactions were discovered in prostate cancer cells and did not focus on their role in metabolic pathways in PCOS or females. SREBPs, comprising SREBP1a, 1c, and 2 isoforms, regulate the expression of most of the enzymes required for lipid synthesis and uptake, making them master regulators of lipid homeostasis. The interactions between androgens and SREBPs are complex and not fully understood. Androgens augment the expression and activation of SREBPs (Heemers *et al.* 2006). This is believed to be indirectly mediated by the transcriptional activity of AR to increase SREBP activation via proteolytic cleavage by SCAP (SREBP cleavage-activating protein) (Heemers *et al.* 2006).

Here, we showed that DHT regulated hepatic lipid metabolism via SREBP1-mediated increased expression of lipogenic genes and proteins resulting in increased hepatic lipid content.

## Materials and methods

### Low-dose DHT animal model

Female C57BL6 mice were fed regular chow and implanted with 4 mm DHT pellets at 2 months of age as described previously (Andrisse *et al.* 2017, 2018, Xue *et al.* 2018) with the exception being that animals were sacrificed at 1-month post insertion instead of 3 months. Empirical data from preliminary studies revealed that metabolic dysfunction was apparent at 1-month post-DHT insertion. The 4 mm DHT pellets contained 2.0 mg of DHT and resulted in a two-fold increase of serum DHT compared to controls. Other groups have used pellets containing 2.5 mg of DHT from Innovative Research of America, which resulted in a six-fold increase compared to controls (Van Houten *et al.* 2012, Van Houten & Visser 2014). As seen previously, the body mass of control and DHT mice were similar at the time of being sacrificed (Con: 29 ± 1; DHT: 30 ± 2; *n* = 6 per group). Mice were fasted overnight as previously done (Andrisse *et al.* 2017) and followed standard fasting guidelines (Ayala *et al.* 2010, Jensen *et al.* 2013). All animal procedures were approved by Howard University’s Institutional Animal Care and Use Committee.

### Histological hepatic lipid content

Using hepatic tissue from overnight-fasted control and DHT mice, lipid content was assessed in hematoxylin and eosin (H&E)-stained and oil red O-stained 5 μM sections following a previously established protocol (Cui *et al.* 2017). The stained slides were assessed via light microscopy and digital image analysis (ImageJ).

### Biochemical hepatic lipid content

Liver samples from overnight-fasted control and DHT mice were harvested, homogenized, and equal amounts underwent a triglyceride (TG) content assay following the manufacturer’s instructions (Sigma Aldrich, Catalog Number TR0100). The TG Determination Kit is for the quantitative enzymatic measurement of glycerol, true triglycerides, and total triglycerides at 540 nm.

### Protein processing and Western blots

Liver tissues from female mice fasted overnight were extracted and analyzed for total protein content with the bicinchoninic acid (BCA) protein assay. Proteins were prepared for Western blot analysis first with separation via sodium dodecyl sulfate PAGE (SDS-PAGE) and transferred to nitrocellulose membranes. They were blocked then incubated with primary antibodies (SREBP1, SREBP2, p-ACC, ACC, FAS, SCAP, ChREBP, FATP1/2, PPARγ, FXR, LXR, and/or pre-B cell colony enhancing factor (PBEF); varying dilution; Table 1), using GAPDH and actin as a cytosolic loading control and TBP as a nuclear loading control. All antibodies were from Santa Cruz Biotechnology. After incubation with secondary antibodies (goat anti-mouse IgG; 1:5000 dilution), enhanced chemiluminescence was used for detection. Densities were quantified via myImage Analysis software (Thermo Scientific) and analyzed by two-tailed *t*-tests with Prism software.

**Table 1:** Antibodies used in this study.

**Peptide/Protein target**	**Antibody ID (RRID)**	**Name of antibody**	**Antibody manufacturer, catalog No.**	**Animal in which antibody was raised; monoclonal or polyclonal**	**Dilution**
Fatty acid synthase	AB_627584	FAS (G-11)	Santa Cruz Biotechnology, no. 48357	Mouse; monoclonal	1:1000
Phospho-acetyl-CoA carboxylase	AB_10710517	pACCα (F-2)	Santa Cruz Biotechnology, no. 271965	Mouse; monoclonal	1:1000
Acetyl CoA carboxylase	AB_2219400	ACCβ (H-7)	Santa Cruz Biotechnology, no. 390344	Mouse; monoclonal	1:1000
SREBP cleavage-activating protein	AB_628237	SCAP (9D5)	Santa Cruz Biotechnology, no. 13553	Mouse; monoclonal	1:1000
Sterol regulatory element-binding protein-1	AB_628282	SREBP1 (2A4)	Santa Cruz Biotechnology, no. 13551	Mouse; monoclonal	1:1000
Sterol regulatory element-binding protein-2	AB_2194250	SREBP2 (1C6)	Santa Cruz Biotechnology, no. 13552	Mouse; monoclonal	1:1000
Carbohydrate-responsive element-binding protein	AB_2146396	ChREBP (G-12)	Santa Cruz Biotechnology, no. 515922	Mouse; monoclonal	1:500
Acyl-CoA synthetase very long chain 1 (FATP2)	AB_2190625	ACSVL1 (D-7)	Santa Cruz Biotechnology, no. 393906	Mouse; monoclonal	1:500
Acyl-CoA synthetase very long chain 5 (FATP1)	AB_1714120	ACSVL5	Abnova Cat# H00376497-D01P	Rabbit Polyclonal	1:500
Peroxisome proliferator-activated receptor gamma	AB_1128606	PPARγ (8D1H8H4)	Santa Cruz Biotechnology, no. 81152	Mouse; monoclonal	1:1000
Farnesoid X receptor	AB_628039	FXR (D-3)	Santa Cruz Biotechnology, no. 25309	Mouse; monoclonal	1:500
Liver X receptor	AB_10611071	LXRα/β (G-10)	Santa Cruz Biotechnology, no. 271064	Mouse; monoclonal	1:1000
Pre-B cell enhancing factor	AB_2251220	PBEF	Santa Cruz Biotechnology, no. 130058	Mouse; monoclonal	1:500
Stearoyl-CoA desaturase	AB_2254143	SCD (D-5)	Santa Cruz Biotechnology Cat# sc-23016	Mouse; monoclonal	1:1000
Glyceraldehyde phospho-dehydrogenase	AB_641103	GAPDH	Santa Cruz Biotechnology Cat# sc-20356	Mouse; monoclonal	1:5000
Caveolae	AB_1120056	Caveolin-1 (4H312)	Santa Cruz Biotechnology, no. 70516	Mouse; monoclonal	1:1000
Fatty acid-binding proteins	AB_10650265	A-FABP (B-4)	Santa Cruz Biotechnology, no. 271529	Mouse; monoclonal	1:1000
TATA-binding subunit of TFIID	AB_1249762	TBP (A-6)	Santa Cruz Biotechnology no. sc-74595	mouse monoclonal	1:2000
Cyclophilin A	AB_2169131	CyPA (6-YD13)	Santa Cruz Biotechnology no. sc-134310	mouse monoclonal	1:1000

### Nuclear and cytoplasmic extraction

The following was performed on the liver lysates: the cell pellet was suspended in a hypotonic buffer (cytoplasmic extraction buffer: 10 mM HEPES, 1.5 mM MgCl2, 10 mM KCl, 0.5 mM DTT, 1 mM EDTA, 0.05% NP40, pH 7.9). Detergent (NP40) was added and vortexed to separate the nuclei from the cytoplasmic fraction. The solution was centrifuged and the supernatant was collected (which contained the cytoplasm). The pellet (containing nuclei) was resuspended in a nuclear extraction buffer (nuclear extraction buffer: 5 mM HEPES, 1.5 mM MgCl2, 4.6 M NaCl, 0.2 mM EDTA, 0.5 mM DTT, 26% glycerol, pH 7.9) that busted the nuclear membrane. It was then centrifuged at high speed and the supernatant (nuclear fraction) was collected. The final pellet was discarded. TBP was used as a nuclear fractionation loading control.

#### Hepatocyte cell line culture with DHT

The serum DHT concentration of the low-dose DHT mouse model was replicated in cell culture as previously described (Han *et al* 2019). Briefly, serum DHT concentrations of low-dose DHT mice were roughly 200 pg/mL (two-fold that of control), which is equal to 1.02 nM. H2.35 female mouse hepatocytes were cultured as previously described (Han *et al* 2019). Cell line authentication was performed by ATCC using STRS analysis. Experiments were performed between three to five cell passages. H2.35 cells displayed a low concentration of AR. AR was transiently transfected as done before (Han *et al* 2019) using Lipofectamine 2000 (Thermo Fisher) with a pSV Sport SREBP-1c vector containing an SREBP1c overexpression vector or a pSV-Sport empty vector (Addgene, Cambridge, MA). Cells were pretreated for 30 min with 10 μmol/L mifepristone (Li *et al.* 2004) (glucocorticoid receptor inhibitor, M8046 Sigma Aldrich), 100 nm enzalutamide (androgen receptor DBD inhibitor, VPC-14449, Sigma Aldrich) (Spaanderman *et al.* 2018) and/or 10 μmol/L Betulin (Yin *et al.* 2019, Lee *et al.* 2020) (Med Chem Express Corporate; NJ, USA, Cat. No. HY-N0083) before fresh media with or without 1 nM DHT was subsequently added for 24 h. Serum starvation was performed for the final 3 h of DHT treatment. BioRad cell lysis was used to harvest the cells, and the lysate was subsequently processed for Western blotting.

### RNA processing and qRT-PCR

RNA was extracted from liver tissues via the Trizol Method. After RNA precipitation, the pellet was re-dissolved with elution buffer, then reverse transcribed by an iScript cDNA kit (Bio-Rad). qRT-PCR was performed using SYBR Green (BioRad) and primers for GAPDH, FAS, ACC, SREBP1, and SCD. See Table 2 for primer sequences. The data are presented at the delta delta Ct-fold change relative to controls.

**Table 2:** qRT-PCR primer sequences.

**qRT-PCR primer sequences**	**Sense (5’ to 3’)**	**Antisense (5’ to 3’)**	**Function**
Sterol regulatory element binding protein 1* (Srebp1)*	TGACCCGGCTATTCCGTGA	CTGGGCTGAGCAATACAGTTC	*de novo* lipogenesis (+)
Fatty acid synthase* (Fas; Fasn)*	GCTGCTGTTGGAAGTCAGC	AGTGTTCGTTCCTCGGAGTG	Fatty acid synthesis (+), lipogenic enzyme
Acetyl co-A carboxylase 1 or alpha* (Acc1; Acaca)*	GGCTCAAACTGCAGGTATCC	TTGCCAATCCACTCGAAGA	Fatty acid synthesis (+), lipogenic enzyme
*Stearoyl-CoA desaturase (Scd)*	TTCCCTCCTGCAAGCTCTAC	CAGAGCGCTGGTCATGTAGT	Fatty acid synthesis, lipogenic enzyme
*Glyceraldehyde phospho dehydrogenase (Gapdh)*	AGGTCGGTGTGAACGGATTTG	GGGGTCGTTGATGGCAACA	Housekeeping gene
*Srebp1* promoter region	TAAGAGCTCGGTACCTCCCCTAGGGC	GGGCCAGGAGTGGGTAAA-	Promoter region; 5*-flanking region of the mouse SREBP-1c gene (Amemiya-Kudo *et al.* 2000)
SREBP cleavage-activating protein (SCAP Intron 8)	CCATACCTGGTGGTCGTTATTG	GAACAGCATCTGGAGGAAGAAG	Intron 8 androgen response element (Heemers *et al.* 2004)

### Immunoprecipitation

Liver and adipose tissue lysates were incubated with SREBP1, SREBP2, or SCAP antibodies (Santa Cruz Biotechnology) overnight at 4°C, and then incubated in protein A/G sepharose beads (Sigma) overnight at 4°C. The samples were micro centrifuged, washed with lysis buffer five times, and re-dissolved in 4× Laemmli sample and dithiothreitol. Then the samples underwent SDS-PAGE and immunoblotting analysis. They were immunoblotted with SREBP1, SREBP2, SCAP, FxR or LxR antibodies (Santa Cruz Biotechnology).

#### Chromatin immunoprecipitation

Chromatin immunoprecipitation (ChIP) was performed using the ChIP-IT Express kit as indicated by the manufacturer (Active Motif, Carlsbad, CA). Briefly, liver tissue lysates were cross-linked with formaldehyde, digested via enzyme and sonication, immunoprecipitated using AR or IgG antibodies, treated with proteinase K, and DNA was isolated and then analyzed via qRT-PCR. The primers are listed in Table 2. The data are presented as percentages of the total input DNA.

### Data and resource availability

The datasets generated during and/or analyzed during the current study are available from the corresponding author upon reasonable request. The resources used and/or generated during and/or analyzed during the current study are available from the corresponding author upon reasonable request. Antibody RRIDs are included in Table 1 and primer sequences are included in Table 2.

### Statistical analysis

Findings were evaluated with two-tailed *t*-tests or one-way ANOVA with Tukey’s multiple comparison tests was used for figures where three or more groups were compared. GraphPad Prism software was used for all statistical analyses. All results were expressed as scatter plots with individual values and mean ± s.e.m., where a value of *P* < 0.05 was defined as statistically significant.

## Results

### Low-dose DHT produced a normal weight PCOS-like mouse model

**Figure 1:**
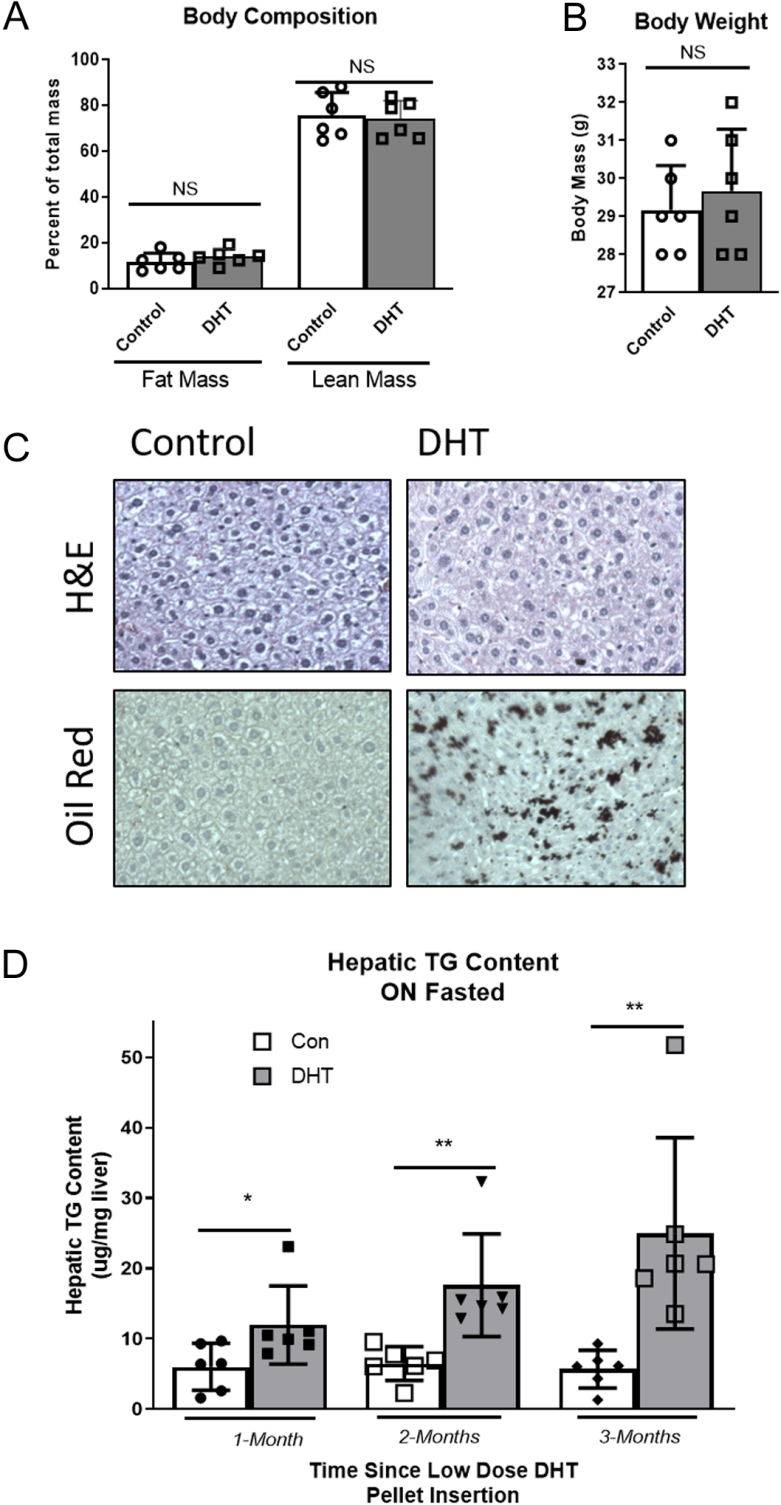
Low-dose DHT (a mouse model of normal weight PCOS) caused NAFLD. (A) Fat mass and lean mass were determined via echo MRI at 1 month after insertion (*n* = 6 per group). (B) Mice were weighed after DHT insertion up until the time they were killed (*n* = 6 per group). (C) After 3 months of low-dose DHT, the hepatic lipid content of DHT mice was significantly higher than that of control mice, as measured by oil red o staining of 5 μM sections of hepatic tissue (*n* = 6 per group).(D) A TG assay kit from Sigma Aldrich was used to determine the hepatic TG content in liver samples from control and DHT mice in months 1, 2, and 3 after control or DHT pellet insertion. *n* = 6 per group. *P < 0.05, **P < 0.01. TG, triglyceride.

Unlike many other PCOS-like rodent models (Van Houten *et al.* 2012, Van Houten & Visser 2014, Stener-Victorin *et al.* 2020), where the PCOS-like animals displayed increased body mass, low-dose DHT mice had the same body composition (lean and fat mass) and body weight as controls (Fig. 1A and B).

To examine if sex steroid and gonadotropin serum hormone levels were altered, DHT, testosterone, estradiol, luteinizing hormone (LH), and follicle-stimulating hormone (FSH) were measured. DHT mice displayed a two-fold increase in serum DHT compared to controls (Supplementary Fig. 1A, see section on supplementary materials given at the end of this article). Testosterone and estradiol were not changed in DHT compared to control mice (Supplementary Fig. 1B and C). LH and FSH serum levels were unchanged in DHT compared to control mice (Supplementary Fig. 1D and E); however, the LH:FSH ratio was increased (Supplementary Fig. 1F) as is similarly seen in women with PCOS.

### Low-dose DHT (a mouse model of normal weight PCOS) caused NAFLD

To histologically examine if low dose DHT mice displayed higher levels of hepatic lipid content, control and DHT mice liver samples were examined via oil red O for hepatic lipid content. After 3 months of low-dose DHT, the hepatic lipid content of DHT mice was higher than that of control mice (Fig. 1C), as measured by oil red o-staining of 5 μM sections of hepatic tissue.

To biochemically examine hepatic lipid content, liver tissues from control and DHT mice were harvested in the fasted state at 1-month, 2-months, and 3-months post-DHT insertion and then underwent a triglyceride (TG) content assay. Low-dose DHT caused a near three-fold increase in hepatic TG content after 1 month, a near four-fold increase after 2 months, and a near five-fold increase after 3 months (Fig. 1D). As 1 month of DHT was sufficient to result in excess hepatic lipid content, the remainder of the experiments were performed at 1-month post-DHT insertion.

### Low-dose DHT lowered cytosolic SREBP1 and increased SCAP-SREBP1 binding in the liver

**Figure 2:**
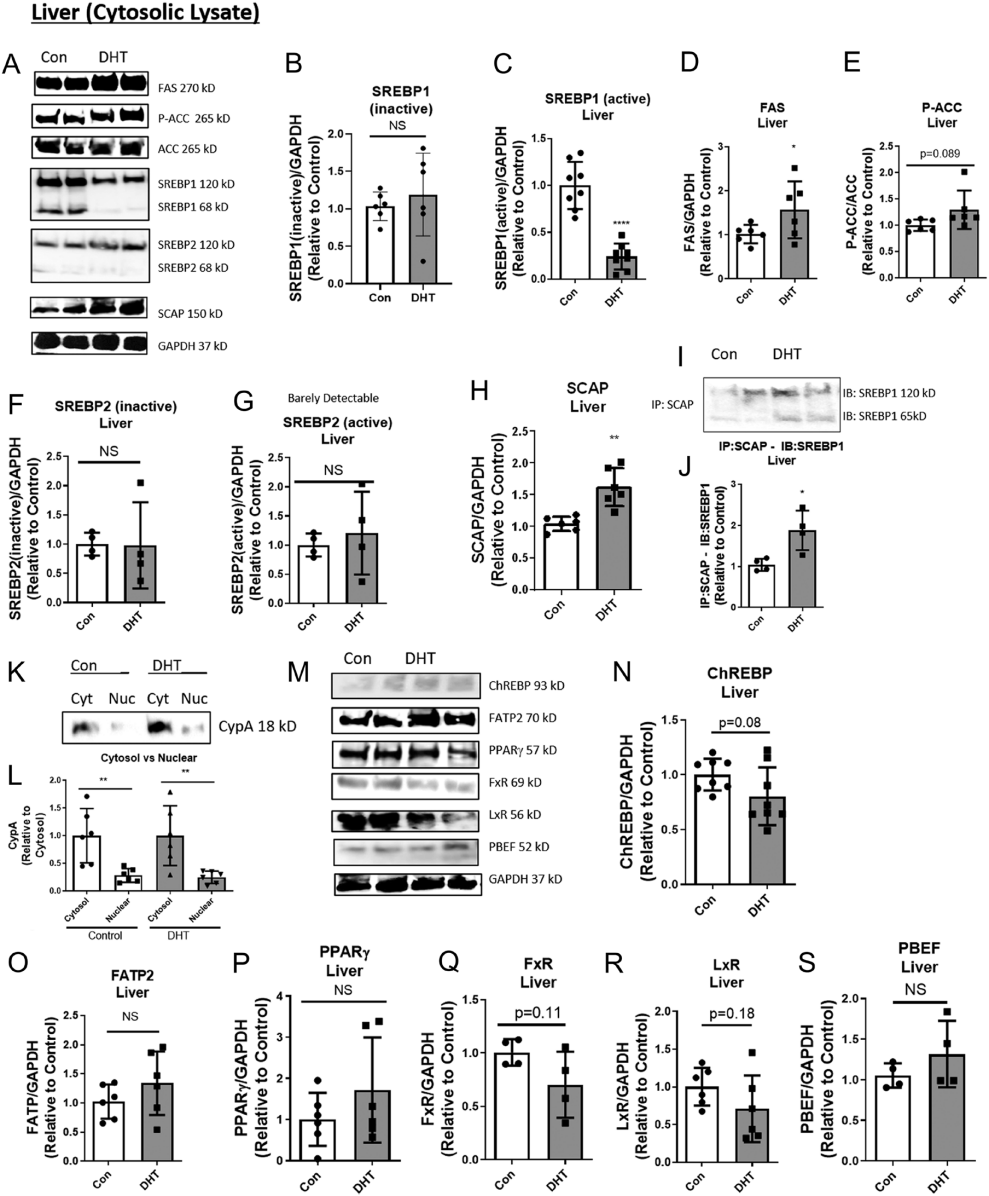
Low-dose DHT lowered cytosolic SREBP1, increased SCAP-SREBP1 binding, and increased FAS in the liver, but did not alter several other pathways involved in lipid metabolism in NAFLD. At 1-month post-insertion, control and DHT mice were fasted for 16 h and livers were harvested, lysed, and subjected to Western blot analysis (A, K, and M) using the following antibodies: (B and C) SREBP1, (D) FAS, (E) p-ACC and ACC, (F and G) SREBP2, (H) SCAP, (N) ChREBP, (O) FATP2, (P) PPARγ, (Q) FxR, (R) LxR, and (S) PBEF. GADPH was used a loading control for cytosolic lysate. (I and J) Liver lysate from above was subjected to an immunoprecipitation experiment using SCAP and blotting for SREBP1. *n*  = 4 to 7 per group, the scatter plot dots represent each individual sample in each group. (K and L) A Western blot using cyclophilin A (CyA) antibodies was used to verify sufficient subcellular fractionation (*n* = 6 per group). One-way ANOVA with Tukey’s multiple comparisons was used for L and unpaired two-tailed *t*-tests were used comparing control to DHT for all other graphs. *P < 0.05, **P < 0.01, ****P < 0.0001. NS equals non-significant. See Table 1 for information on antibodies used for Western blots. FAS, fatty acid synthase; ACC, acetyl-CoA carboxylase; SCAP, SREBP cleavage-activating protein; SREBP1, sterol regulatory element-binding protein 1; ChREBP, carbohydrate-responsive element-binding protein; FxR, farnesoid X receptor; LxR, liver X receptor; PPARγ, peroxisomal proliferator-activated receptor gamma.

To determine if lipogenic proteins played a role in the DHT-induced increased hepatic lipid content, liver lysates of DHT and control mice were examined by Western blot analysis. Low-dose DHT mice displayed no change in the 120 kD (kilodalton) inactive (iSREBP1), decreased 68 kD cleaved forms of cytosolic SREBP1 (Fig. 2A, B and C) and increased levels of FAS (Fig. 2A and D). Inactive and cleaved forms of SREBP2 and p-ACC levels were the same in control and DHT mice (Fig. 2A, E, F and G). Interestingly, DHT increased SCAP protein expression (Fig. 2A and H) and increased SCAP-SREBP1 binding (Fig. 2I and J). Notably, the SCAP-SREBP1 (68 kD) co-IP is likely to be the inactive cleaved form, as SCAP is only known to bind to the c-term inactive region of SREBPs (Brown & Goldstein 1997). Showing increased SCAP binding to the SREBP1 inactive cleaved form may suggest that more of the active cleaved form is present as well. Cyclophilin A (CypA, a cytosolic loading control) expression was more abundant in the cytosol than the nucleus in both control and DHT mice (Fig. 2K and L), suggesting that the cytosol and nuclear sub-fractionation were sufficient.

### Low-dose DHT did not alter other lipid metabolic pathways

To determine if other lipid metabolic pathways played a role in the DHT-induced increased hepatic content, several commonly known lipid pathways were examined in liver. Low-dose DHT did not alter ChREBP (glucose-stimulated DNL), FATP2 (FA uptake), PPARγ (FA synthesis & storage), FxR (DNL inhibition), LXR (DNL activation), and PBEF (hepatic inflammation) compared to controls (Fig. 2M, N, O, P, Q, R and S).

### Low-dose DHT increased nuclear SREBP1

**Figure 3:**
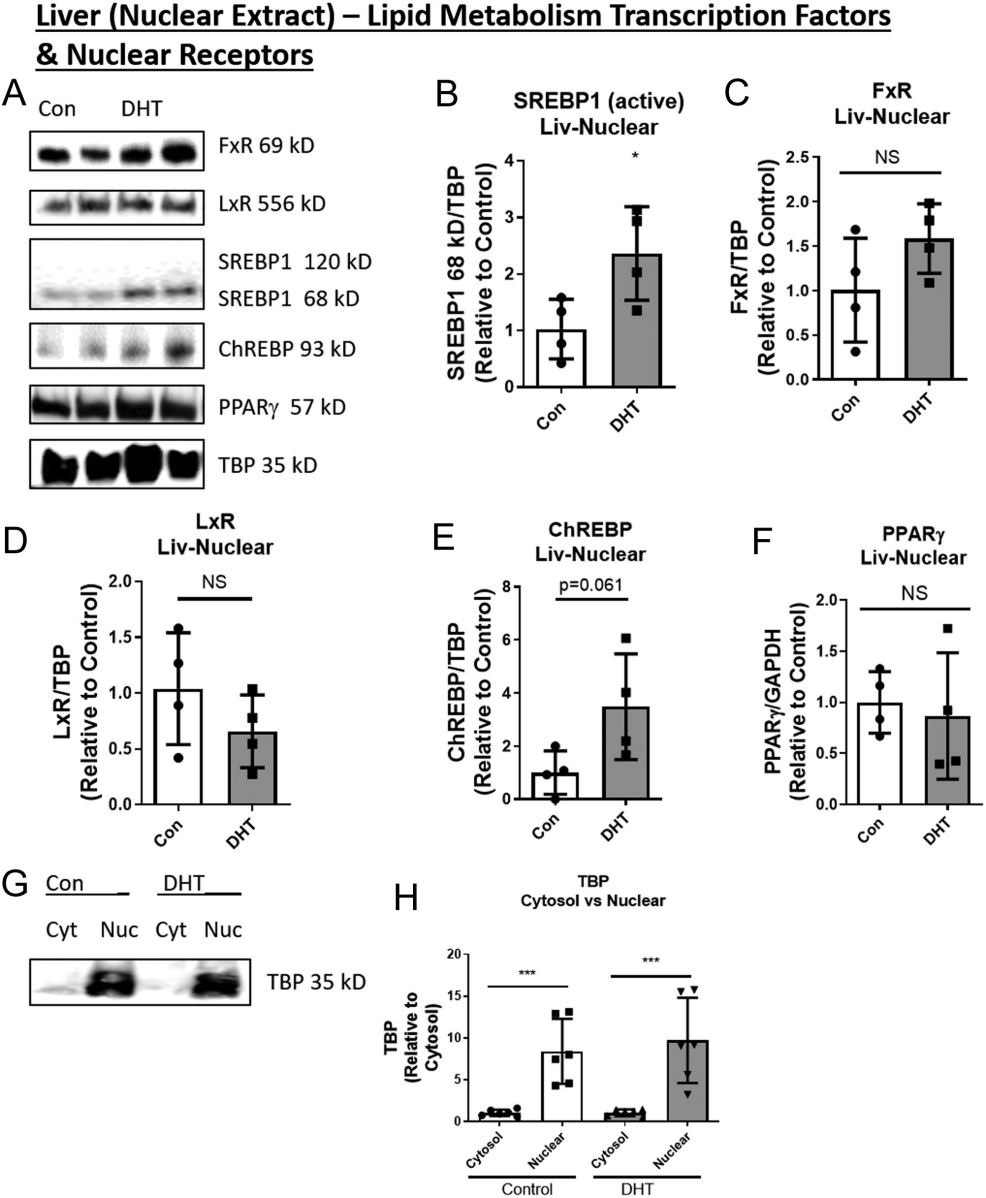
Low-dose DHT increased nuclear SREBP1 but not several other lipid metabolism nuclear proteins. At 1-month post-insertion, control and DHT mice were fasted for 16 h and livers were harvested, underwent nuclear extraction as detailed in the methods, and subjected to (A) Western blot analysis using the following antibodies: (B) FxR, (C) LxR, (D) SREBP1, (E) ChREBP, and (F) PPARγ. (G and H) TBP was used a loading control for nuclear extracts. For the subcellular fractionation control blot, Cyt = cytosol and Nuc = nuclear. *n* = 4 per group for B, C, D, E and F and *n* = 6 per group for H. The scatter plot dots represent each individual sample in each group. One-way ANOVA with Tukey’s multiple comparisons was used for H and unpaired two-tailed *t*-tests were used comparing control to DHT in all other graphs. *P < 0.05, ***P < 0.001. See Table 1 for information on antibodies used for Western blots. ChREBP, carbohydrate-responsive element-binding protein; FxR, farnesoid X receptor; LXR, liver X receptor; PPARγ, peroxisomal proliferator-activated receptor gamma; SREBP1, sterol regulatory element-binding protein 1; NS, non-significant.

Nuclear extractions were performed to determine the expression levels of several lipid metabolism nuclear proteins. Low-dose DHT increased SREBP1 nuclear expression compared to controls (Fig. 3A and B) but did not alter the nuclear expression of FxR, LxR, ChREBP, or PPARγ (Fig. 3A, C, D, E and F). Subcellular Fractionation control blots showed that TATA-binding protein (TBP, a nuclear loading control) was absent from the cytosolic extract but present in the nuclear extract in control and DHT mice (Fig. 3G and H).

### Lipogenic mRNA expression was altered in liver tissues of low-dose DHT mice and a female mouse hepatocyte cell line

**Figure 4:**
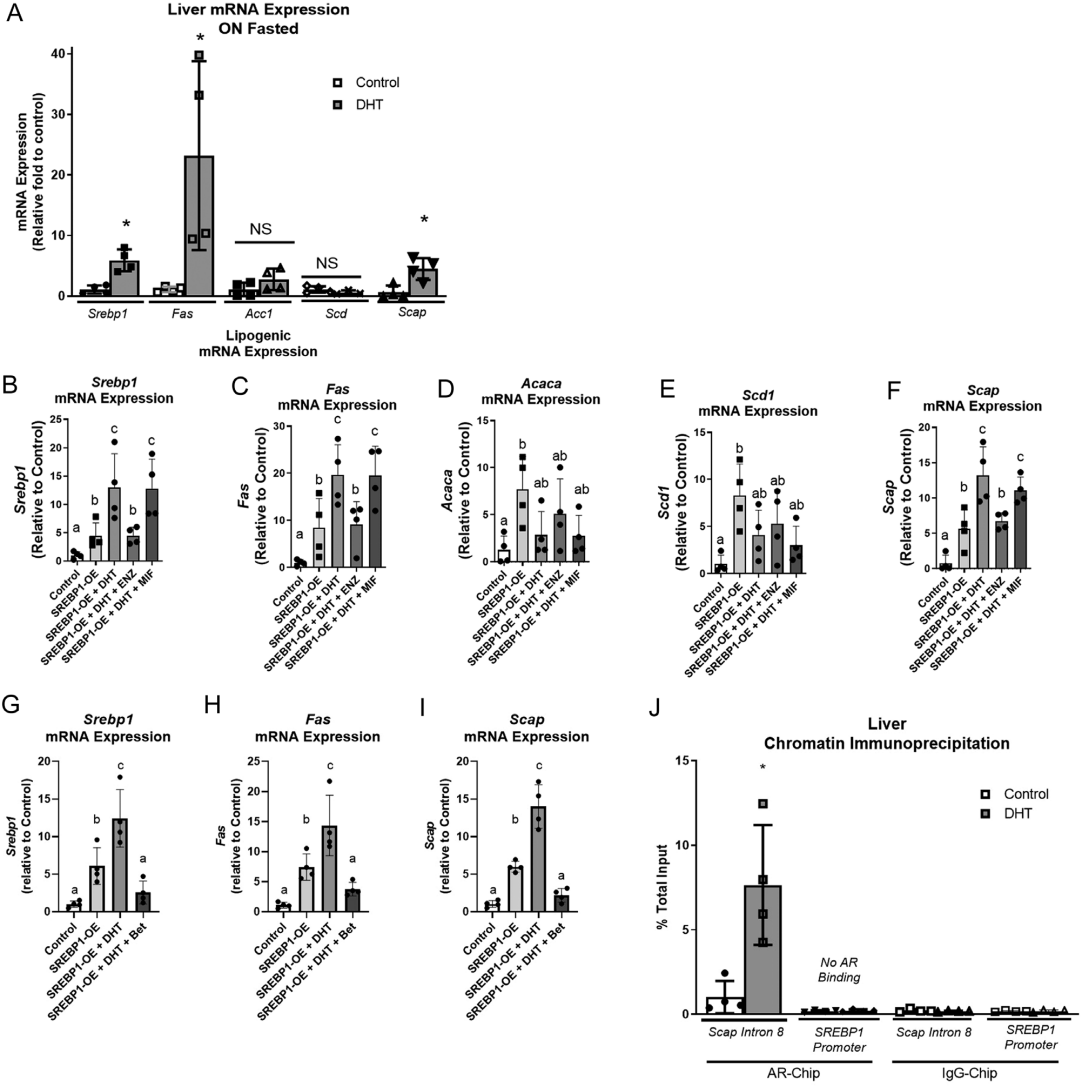
Lipogenic mRNA expression and ChIP analysis in liver tissues of low-dose DHT mice and cell culture. At 1-month post insertion, (A) liver tissues of control and DHT mice were harvested in the fasted state and processed for qRT-PCR analysis using Trizol for RNA isolation. (B, C, D, E and F) H2.35 liver hepatocyte cells transfected with an SREBP1-overexpression (OE) vector were pretreated for 30 min with 10 μmol/L mifepristone (glucocorticoid receptor inhibitor) and/or 100 nM enzalutamide (androgen receptor inhibitor) before fresh media with or without 1 nM DHT was added for 24 h, then serum starved for 3 h, then harvested and processed for qRT-PCR analysis. (G, H and I) SREBP1-OE transfected H2.35 cells were pretreated for 30 min with 10 μmol/L betulin (SCAP inhibitor) before fresh media with or without 1 nM DHT was added for 24 h, then serum starved for 3 h, then harvested and processed for qRT-PCR analysis. (J) Liver tissue from control and DHT mice were harvested in the fasted state and then underwent AR- or IgG-ChIP analysis as described in the methods, probing for *SCAP* Intron 8 or *SREBP1* promoter region. See Table 2 for a list of the abbreviations and functions of qRT-PCR primers. One-way ANOVA with Tukey’s multiple comparisons was used for B, C, D, E, F, G, H , I and J and unpaired two-tailed *t*-tests were used comparing control to DHT. *n* = 4 per group, the scatter plot dots represent each individual sample in each group; *P < 0.05 compared to control, and different letters represent being statistically different from each other. ACC, acetyl-CoA carboxylase; SCAP, SREBP cleavage-activating protein; SREBP1, sterol regulatory element-binding protein 1.

To determine if lipogenic mRNA expression was altered in the low-dose DHT model, liver tissue lysates of control and DHT mice were examined by qRT-PCR analysis. Low-dose DHT mice displayed increased *Srebp1*, *Fas,* and *Scap* mRNA expression in the liver compared to controls (Fig. 4A). *Acc1* and *Scd* mRNA expression in the liver was not changed in DHT mice compared to controls (Fig. 4A).

To determine if this effect was also present in a cell culture model of low-dose DHT where SREBP1 was overexpressed (OE), H2.35 cells (a female mouse hepatocyte cell line) were transiently transfected with SREBP1-OE, incubated with DHT, and examined by qRT-PCR analysis. As expected, SREBP1-OE increased *Srebp1* mRNA expression and the mRNA expression of lipogenic genes, *Fas*, *Acc1*, *Scd*, and *Scap*, (Fig. 4B, C, D, E and F). Similarly, to what was observed in liver tissue, DHT treatment further increased the mRNA expression of *Srebp1*, *Fas*, and *Scap*; but did not alter *Acc1* or *Scd* mRNA expression (Fig. 4B, C, D, E and F).

### Inhibition of glucocorticoid receptor did not alter the DHT-induced increased of SREBP1, SCAP, or FAS

Glucocorticoid receptor (GR) has been shown to bind to SCAP (Heemers *et al.* 2004), and DHT has been shown to activate GR (Spaanderman *et al.* 2018) Thus, to examine if GR and/or AR is mediating the DHT-induced increased lipogenic mRNA expression, we incubated H2.35 hepatocytes in media containing a GR inhibitor, mifepristone, or an AR inhibitor, enzalutamide, with and without DHT, then harvested and analyzed the lysate via qRT-PCR. The GR inhibitor (mifepristone) did not alter the DHT-induced changes; however, the AR inhibitor (enzalutamide) prevented the DHT-induced effect on lipogenic mRNA expression (Fig. 4B, C, D, E and F). These findings suggest that AR and not GR is mediating the DHT-induced alterations in *Srebp1*, *Scap*, or *Fas* mRNA expression.

Additionally, inhibition of SCAP using 10 μmol/L Betulin prevented the DHT-induced increased mRNA expression of *Srebp1*, *Fas*, and *Scap* (Fig. 4G, H and I). Further suggesting that it is DHT’s regulation of SCAP leading to the observed increased lipogenesis and lipid accumulation.

### ChIP analysis in liver tissues of low-dose DHT mice

To examine the mechanism by which low-dose DHT may be altering mRNA expression of *Srebp1* and *Scap*, liver tissues of control and DHT mice underwent AR- and IgG-ChIP analysis of the promoter region of SREBP1 and intron 8 of SCAP. Upon searching the literature, we discovered a novel androgen response element (ARE) residing in intron 8 of SCAP (Heemers *et al.* 2004) and designed a primer using the IDTA Primer Quest Tool that includes intron 8. For the SREBP1 promoter region primer, we used a previously designed primer encompassing the DNA sequence of the 5’-flanking region of the mouse SREBP-1c gene (Amemiya-Kudo *et al.* 2000). See Table 2 for primer sequences. DHT mice displayed a nearly eight-fold higher binding of AR to *Scap* intron 8 than did control mice (Fig. 4E), suggesting that AR binding to intron 8 in *Scap* may play a role in regulating Scap and subsequently SREBP1 mRNA and protein levels. AR binding in the promoter region of SREBP1 was not observed (Fig. 4E). AR inhibitor, enzalutamide, prevented the DHT-induced increased AR binding to SCAP intron 8; whereas, GR inhibitor, mifepristone, did not alter the DHT-induced increased AR binding to SCAP intron 8 (data not shown).

## Discussion

**Figure 5:**
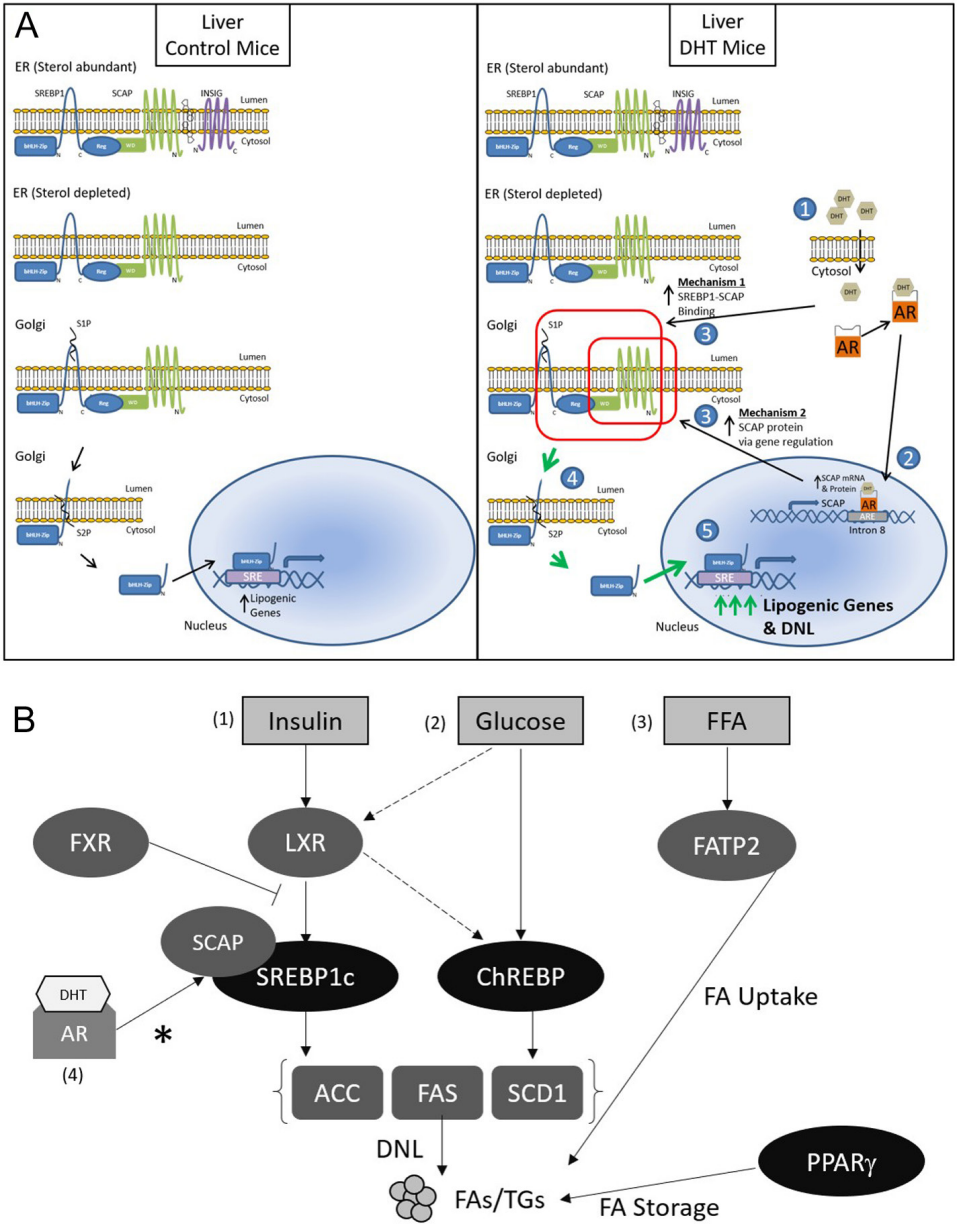
Proposed model: obesity-independent NAFLD in normal weight PCOS-like mouse model. (A) Here we show two proposed mechanisms by which DHT may increase hepatic lipid content. The steps are as follows: *In the liver of control mice* (normal physiology), SREBP1 remains in the ER via binding with SCAP and INSIG. When sterol is low (in the fed state of normal physiology (Leveille, 1969)), SCAP dissociates from INSIG and the SCAP-SREBP1 complex is translocated to the Golgi. Then, proteases, S1P and S2P, cleave SREBP1, releasing the active version of SREBP1 into the cytosol. The active SREBP1 travels to the nucleus, where it activates genes involved in lipid synthesis. *In the liver of DHT mice*, (1) DHT enters the cell, binds, and activates AR, (2) AR enters the nucleus and binds to intron 8 of SCAP leading to increased *Scap* mRNA and subsequently increased SCAP protein levels, (3) increased SCAP protein expression and increased SCAP binding to the 65 kD cleaved inactive SREBP1 leads to, (4) increased nuclear (active) SREBP1 which leads to, (5) increased lipogenic mRNA expression resulting in increased *de novo* lipogenesis. Overall, AR binding to intron 8 in *Scap* may be a mechanism leading to increased SCAP which led to SCAP-SREBP1 binding being elevated which led to increased active nuclear SREBP1 and increased lipogenic gene expression. Key: Larger green arrows indicate greater increase as compared to the thinner black arrows. The red box highlights the proposed mechanism. (B) Transcriptional control of lipogenesis and lipid metabolism in the liver: *de novo* lipogenesis (DNL) is known to be controlled by glucose and insulin signaling pathways, leading to increased expression of lipogenic genes. *What is known*: (1) Insulin stimulates the activity of SREBP1c, a transcription factor that augments lipogenic enzymes (ACC1, FAS, SCD1), via activation of LXR and several other methods not depicted (for more details see Dorotea *et al.* 2020). FXR lowers lipid synthesis by decreasing SREBP1c and LXRα activity, (2) glucose promotes the activity of ChREBP, another transcription factor that increases lipogenic and glycolytic (not depicted) enzymes. ChREBP is directly regulated by LXRs as well and it controls the amount of MUFA-to-SFA, creating more MUFA by activating SCD1. Glucose has also been shown to activate LXR’s genes, (3) free fatty acids (FFAs) from the serum enter the hepatocytes from fatty acid transport protein 2 (FATP2) and are incorporated into lipid droplets with the assistance of PPARγ which stimulates FA storage*. What we discovered here*: (4) We examined the effect of low-dose DHT on all of the genes and proteins depicted in Fig. 5B and none of them were altered by DHT except SCAP, SREBP1, FAS, and ACC. As depicted in Fig. 5A, low-dose DHT increased SCAP mRNA, protein, and binding to SREBP1 leading to increased FAS and ACC leading to increased hepatic lipid content. ACC, acetyl-CoA carboxylase; ChREBP, carbohydrate-responsive element-binding protein; FA, fatty acid; FAS, fatty acid synthase; FFAs, free fatty acids; FxR, farnesoid X receptor; LxR, liver X receptor; MUFA, monosaturated fatty acids; PPARγ, peroxisomal proliferator-activated receptor gamma; SCAP, SREBP cleavage-activating protein; SCD1, stearoyl CoA desaturase 1; SFA, saturated fatty acids; SREBP1c, sterol regulatory element-binding protein 1c; TG, triglyceride.

Here, we show that female mice treated with low-dose DHT developed obesity-independent hepatic steatosis that was supported by increased lipogenic gene and protein expression via regulation of hepatic SREBP1. We showed that DHT increased SCAP and SCAP-SREBP1 binding which resulted in a decrease in active cytosolic SREBP1 and increased active nuclear SREBP1. We propose that increased active nuclear SREBP1 is the driver for increased lipogenic gene expression (*Srebp1* and *Fas*) and the subsequent obesity-independent hepatic steatosis that was observed (Fig. 5A; Proposed model). More specifically, we propose that AR binding to intron 8 in *Scap* may be a mechanism leading to increased SCAP which led to SCAP-SREBP1 binding being elevated which led to increased active nuclear SREBP1 and increased lipogenic gene expression (Fig. 5A, Mechanistic pathway). No other hepatic lipid metabolism pathway that was examined was altered. These findings (depicted by an asterisk in Fig. 5B) add to the already known mechanisms and pathways of SREBP regulation.

### Significance to clinical findings

Previous research has shown that high androgen levels may play a role in the increased rate of NAFLD seen in women with PCOS (Jones *et al.* 2012, Baranova *et al.* 2013, Petta *et al.* 2017, Kumarendran *et al.* 2018, Minato *et al.* 2018, Wu *et al.* 2018, Harsha Varma *et al.* 2019 ). Various studies have taken the association between high androgen levels and increased presence of NAFLD one step further to show that it is independent of BMI (Chen *et al.* 2010, Vassilatou *et al.* 2010, Jones *et al.* 2012, Cai *et al.* 2017, Kim *et al.* 2017, Harsha Varma *et al.* 2019). The findings of this study provide translatable evidence of a potential underlying mechanism to support the clinical studies showing that HA causes NAFLD independent of obesity.

### Potential sources of hepatic lipid accumulation

Lipids obtained from the diet are packaged into chylomicrons and hydrolyzed, liberating FAs resulting in about 20% being carried to the liver (Ekstedt *et al.* 2006). During fasting, decreased plasma insulin arouses adipocyte TG hydrolase, thus discharging FFAs that are brought to the liver. In the liver, FFAs derivatives from (1) peripheral tissue, (2) endogenous synthesis, or (3) food intake can be utilized for: (a) energy production; (b) storage as TGs in lipid droplets; or (c) packaged with apolipoprotein B into VLDL that is released into the circulation (Kawano & Cohen 2013). The conundrum of NAFLD is that hepatic lipid content via uptake (peripheral or diet) and DNL are not compensated by FA oxidation or construction of VLDL particles. Low-dose DHT mice exhibited obesity-independent NAFLD. As such, here, we examined key players in lipid content uptake and DNL.

### Discussion of low-dose DHT regulating lipid metabolism in the liver

Lipid metabolism is regulated by a multitude of pathways and key proteins and genes. In the following paragraphs, we discuss a few of these key players. ChREBP is required for the induction of glycolytic gene expression by glucose, and it acts together with SREBP1c to stimulate lipogenic genes (Dentin *et al.* 2004). SREBP1c activity alone has been shown to be insufficient for the full stimulation of glycolytic and lipogenic gene expression (Linden *et al.* 2018). Here, we saw no difference in cytosolic or nuclear ChREBP levels in control and DHT mice.

FATP2, primarily expressed in the liver and kidney, is unique to other FATPs in that it has been shown to function as both a fatty acid transporter and an acyl-coenzyme A synthetases (ACS) (Krammer *et al.* 2011). Interestingly, here, we saw no alteration in FATP2 levels.

Increased PPARγ expression has been found in steatotic livers (Okamura *et al.* 2010). Thus, it has been suggested that the role of PPARγ in the activation of lipogenic genes may contribute to the development of steatosis. Here, we observed no change in cytosolic or nuclear PPARγ in control compared to DHT mice.

FxR is mainly expressed in the liver, intestine, kidneys, and adrenal glands, with less expression in adipose tissue and the heart (Zhang *et al.* 2003). FxR activation seems to reduce TG levels by: (1) reducing FA synthesis in the liver, through the reduction of SREBP1c and LxR expression (Yang *et al.* 2010); (2) inducing the expression of PPARα, which promotes FFA catabolism via β-oxidation; (3) increasing TG clearance; and (4) increasing adipose tissue storage and altering adipokine patterns (Teodoro *et al.* 2011). Here, we saw no difference in cytosolic or nuclear FxR or LxR in control compared to DHT mice.

Thus, DHT-induced increased hepatic lipid content was not due to FATP (increased FA uptake or increased FA activation), PPARγ (increased FA storage), FxR (decreased inhibition of SREBP1), LxR (increased upstream activation of SREBP1), or PBEF (increased hepatic inflammation).

### Discussion of other studies examining the effect of DHT on hepatic lipid metabolism

One study (O'Reilly *et al.* 2017) described an intra-adipose mechanism of androgen activation that contributes to adipose remodeling and a systemic lipotoxic metabolome, with intra-adipose androgens driving lipid accumulation in PCOS. This paper is primarily a human study and, moreover, does not describe the effects of androgens on liver DNL. Moverare-Skrtic *et al.* (2006) showed that DHT-induced AR activation resulted in obesity and altered liver lipid metabolism in orchiectomized mice. This study was in male mice, not female mice.

In the supplemental data of a previous study by the PI of this laboratory (Andrisse *et al.* 2017), low-dose DHT did not increase histological hepatic lipid content in fed mice. In contrast, for the current study, histological and biochemical hepatic lipid content was assessed in mice that were fasted overnight. In the fed state, insulin levels are drastically increased (Feldman & Lebovitz 1970) and cholesterol levels are significantly reduced (Leveille 1969) compared to the fasted state. Both result in increased SREBP1 activity. Insulin activates SREBP1 and increases lipid synthesis in normal physiology (Nadeau *et al.* 2004, Dif *et al.* 2006); and when cholesterols are low, INSIGs are rapidly degraded allowing the SCAP-SREBP complex to move from the ER to the Golgi beginning the SREBP activation process (Gong *et al.* 2006, Dorotea *et al.* 2020). Thus, in the fed state, it may be more difficult to observe small increases in lipid synthesis. In the fasted state (with no insulin stimulation and higher cholesterol), there will be little to no physiological insulin-stimulated lipid synthesis; thus, making small pathophysiological changes more observable. Additionally, in the previous study (Andrisse *et al.* 2017), the DHT and control mice were injected with insulin and sacrificed 10 min later to assess insulin action. In this study, insulin signaling was not the primary focus, thus the mice were not injected with insulin.

### Limitations

In Fig. 2A, the cleaved form of SREBP1 was detected in the cytosolic lysate of hepatocytes. Since it has been well-established by Brown and Goldstein that the processed (or active) form of SREBP1 moves to the nucleus and activates transcription of the target genes (Brown & Goldstein 1997), it is difficult to interpret the existence of active SREBP1 protein in the cytosolic lysate. S1P cleaves the 120 kD form of SREBP1 nearly in half in the Golgi (Kim *et al.* 2018). It is not until the second cleavage by S2P that active SREBP travels to the nucleus (Rawson *et al.* 1997). Thus, there is a period of time that a roughly 68 kD form of SREBP1 will be present in the cytosol. This may be what we are detecting here in this study. To our knowledge, there is not an SREBP1 antibody that can distinguish between the S1P cleaved from and the S2P cleaved form.

### Future studies

INSIG-1 and INSIG-2 perform important functions in the negative-feedback system for cholesterol production (Han *et al.* 2019). INSIG-1 has high expression in the liver and is found in the endoplasmic reticulum (ER), where it binds the sterol-sensing domain of SREBP cleavage-activating protein (SCAP) (Han *et al.* 2019). Sterol stimulates INSIG-1 binding to SCAP. INSIG-2, also an ER protein, binds SCAP in a sterol-regulated fashion. Notably, INSIG-1 and INSIG-2 prevent the transfer of SCAP from the ER and thus thwart cholesterol synthesis by blocking the proteolytic processing of SREBPs in the Golgi (Han *et al.* 2019). Future studies should examine if DHT alters INSIG-1, INSIG-2, S1P, and S2P. Additionally, assessing how the DHT-induced lipogenic regulation discovered here is impacted by insulin action and how it might fit into the hepatic insulin resistance paradox (Santoleri & Titchenell 2019) is an area for future examination.

### Overall conclusion

Our findings support evidence that low-dose dihydrotestosterone increased *de novo* lipogenic proteins resulting in increased fatty liver via regulation of SREBP1 in the liver from normal weight female mice. It is generally known that DHT enters the cell and binds with and activates AR. We show that in the presence of low-dose DHT the interaction between SCAP and SREBP1 is increased leading to increased nuclear aSREBP1 resulting in increased DNL. We propose that the mechanism of action may be increased DHT-induced AR binding to an ARE in SCAP intron 8.

### Supplementary Material

Suppl. Fig. 1

## Declaration of interest

The authors declare that there is no conflict of interest that could be perceived as prejudicing the impartiality of the research reported.

## Funding

This work was supported by Dr Andrisse’s Howard University Faculty startup funds. Technical support in the form of summer research fellowships to Patrick McWhorter and Jessie Myer was provided by American Physiological Society’s Undergraduate Summer Research Fellowship; and Alpha Omega Alpha Carolyn L. Kuckein Student Research Fellowship to Tina Seidu. Dr Stanley Andrisse is the guarantor of this work and, as such, had full access to all the data in the study and takes responsibility for the integrity of the data and the accuracy of the data analysis.

## Data availability

The datasets generated during and/or analyzed during the current study are not publicly available but are available from the corresponding author on reasonable request.

## Author contribution statement

S A conceived and designed the research; all authors performed one or more experiments; and all authors analyzed one or more data sets; S A interpreted the results of the experiments; all authors prepared one or more figures; S A drafted the manuscript; T S, P M, and J M had equal contributions of authorship. S A and T S edited and revised the manuscript; all authors approved the final version of manuscript.
