# Supplementary material for: DHT causes liver steatosis via transcriptional regulation of SCAP in normal weight female mice
Source: J Endocrinol. 2021 Jun 1;250(2):49–65. doi: 10.1530/JOE-21-0040 (PMC8240729; doi:10.1530/JOE-21-0040)
Supplement: Suppl. Fig. 1 [file supplementary_figure_1.pdf]

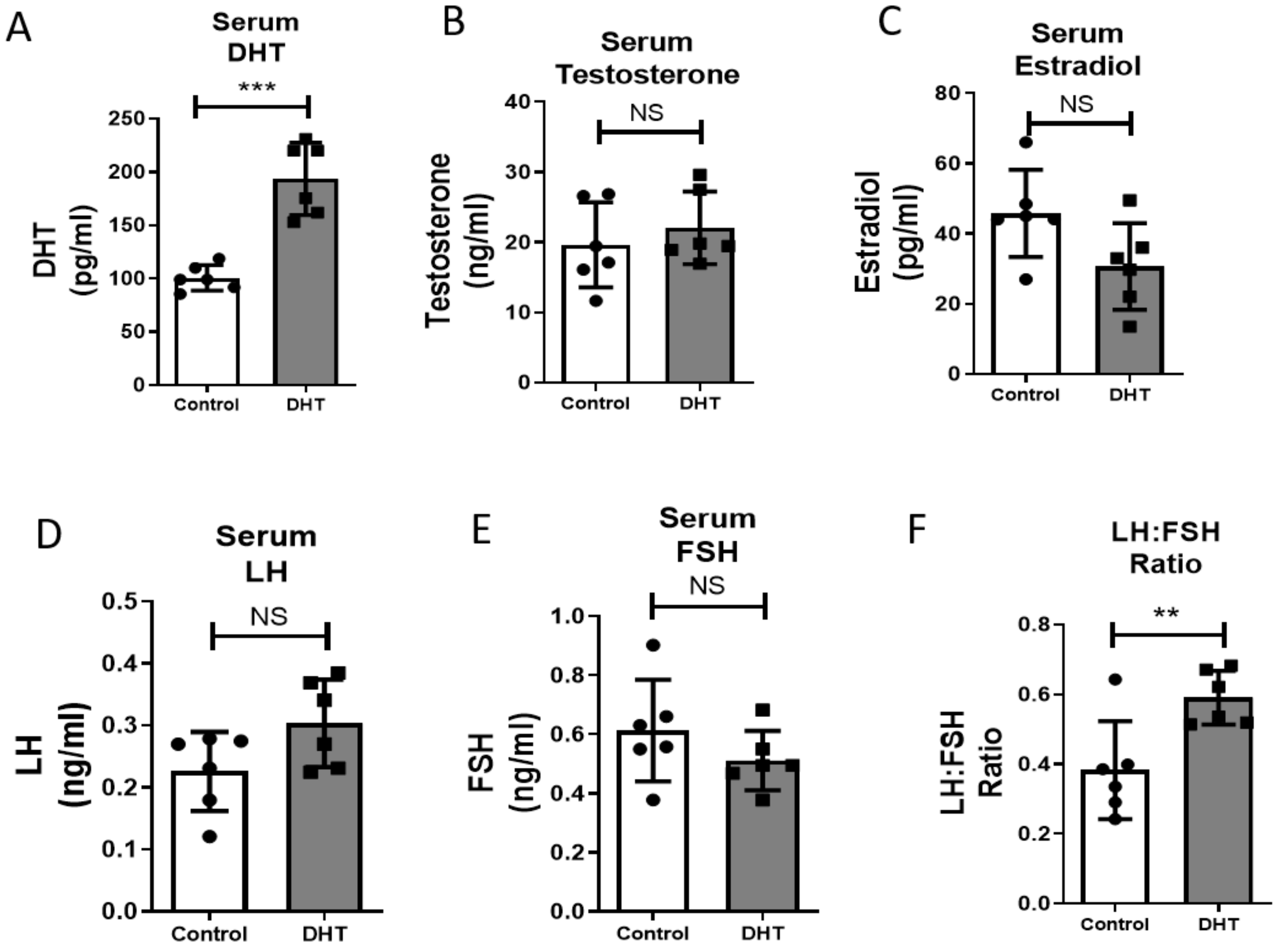

Supplemental Figure 1. Sex steroid and gonadotropin hormone levels. A) DHT levels from control and DHT mice were measured weekly for 30 days post insertion by enzyme-linked immunosorbent assay (ELISA) (Alpha Diagnostics International, San Antonio, TX). B-C) Serum levels of testosterone and estradiol from control and DHT mice were measured at 1-month post insertion via ELISA (Abcam, Cambridge, United Kingdom; catalog # ab108666 and # ab108667, respectively). D-F) Morning levels (9 to 10 am) of luteinizing hormone (LH) and follicle stimulating hormone (FSH) from serum of control and DHT mice at diestrus at 1-month post insertion were measured by ELISA (Novus Biologics, Littleton, CO; USA; catalog # KA2332 and # KA2330, respectively).
